# Towards ending viral hepatitis as a public health threat: translating new momentum into concrete results in South-East Asia

**DOI:** 10.1186/s13099-018-0237-x

**Published:** 2018-03-05

**Authors:** Poonam Khetrapal Singh

**Affiliations:** World Health Organization, Regional Office for South-East Asia, World Health House, Indraprastha Estate, Mahatma Gandhi Marg, New Delhi, 110 002 India

We are living through a “golden age” of global health with respect to communicable diseases. As a result of scientific breakthroughs, global solidarity and focused funding, mortality associated with leading communicable diseases is on the decline. Indeed, since 2000, the progress in combatting the leading communicable diseases (HIV, tuberculosis and malaria) has prompted the global community to commit to end these epidemics once and for all by 2030.

But there is one glaring exception to the good news regarding communicable diseases. While mortality from HIV, Tuberculosis, and Malaria is now declining, mortality caused by viral hepatitis is on the rise. The viral hepatitis challenge is monumental, as it is the seventh leading cause of death worldwide. Annual deaths from hepatitis (1.34 million) [1] exceed the number of AIDS-related deaths (1 million) [2] and approach mortality associated with tuberculosis (1.67 million) [3].

The continued rise in hepatitis-related deaths is both alarming and ironic, as hepatitis is wholly preventable and, in the case of hepatitis C, curable. Although viral hepatitis is a major global health challenge, the world has yet to bring to the fight against hepatitis the seriousness, passion and focus that we have seen for other leading communicable diseases.

However, there is now reason to believe that the global community is ready to take viral hepatitis seriously. In 2014, the World Health Assembly called on WHO to develop a global strategy for viral hepatitis. Two years later, WHO Member States unanimously endorsed the WHO Global Health Sector Strategy for Viral Hepatitis 2016–2021 [4]. This global strategy aims to achieve for viral hepatitis what the world now seeks for other leading communicable diseases—eliminating hepatitis as a public health threat by reducing new infections by 90% and mortality by 65% by 2030 (Fig. 1).

**Fig. 1:**
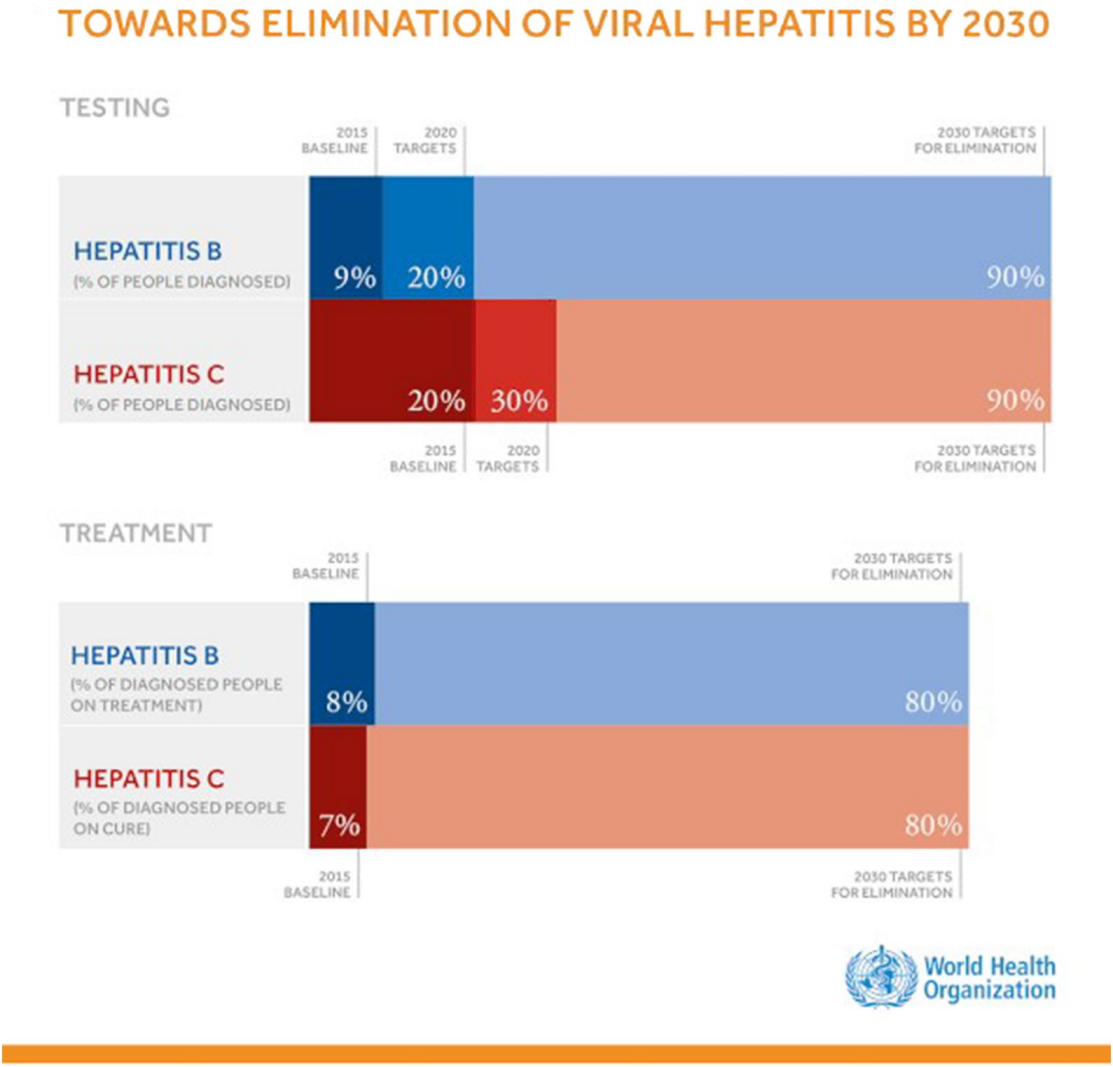
Global 2020 and 2030 targets for hepatitis elimination (Source: Global Hepatitis Report 2017 accompanying graphic at http://www.who.int/hepatitis/news-events/global-hepatitis-report2017-infographic/en/. Accessed on 5 February 2018)

## Hepatitis globally and in South-East Asia: a status report

Viral hepatitis is caused by a series of different viral types. The most serious are hepatitis B and C, which together cause around 96% of hepatitis related deaths worldwide. An estimated 257 million people globally are infected with hepatitis B virus (HBV), and roughly 900,000 die every year of HBV [1]. It is estimated that 71 million people around the world are infected with hepatitis C virus (HCV) and that 400,000 people die of HCV-related causes each year [1].

Viral hepatitis is a worldwide problem but one that affects Asia the most of all. The WHO South-East Asia Region is home to an estimated 39 million people with chronic HBV and an estimated 10 million people with HCV [1]. An estimated 410,000 people in the region die annually due to viral hepatitis, with chronic complications associated with HBV and HCV accounting for 78% of the total [5].

Hepatitis A and E, water-and foodborne infections, have largely been controlled due to improvements in water, sanitation and hygiene. Yet, multiple outbreaks of these preventable diseases continue to be reported from across the region. Outbreaks of waterborne and foodborne hepatitis due to hepatitis viruses A and E continue to be reported from countries in the region, and there are an estimated 5416 and 31704 deaths per year attributed to hepatitis A and E, respectively [5].

**Fig. 2:**
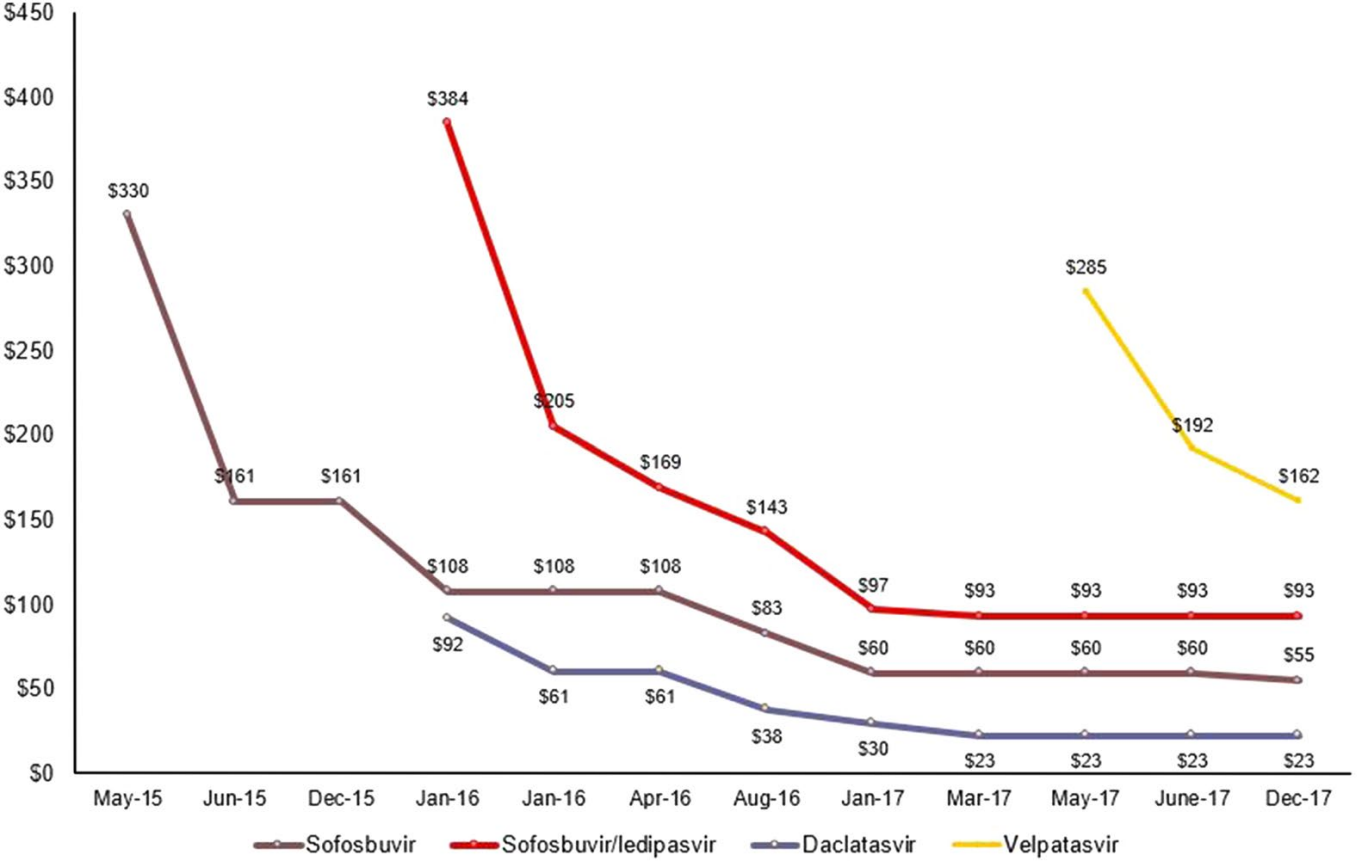
Declining prices for DAAs. Note: All figures are in US$ (Source: TREAT Asia/amfAR-The Foundation for AIDS Research, Bangkok, Thailand)

Much has been accomplished in developing effective strategies and tools to prevent and treat viral hepatitis. In recent years, direct-acting antivirals have emerged for the treatment of hepatitis C, and persons who receive these treatments are cured up to 95% of the time [6]. Over the last 2 years, prices for these breakthrough regimens have plummeted, offering hope for much swifter progress to prevent HCV-related deaths (Fig. 2) [7]. The availability of effective preventive and therapeutic tools underscores the need to ensure universal access among people at risk for or living with viral hepatitis.

## Leveraging a new momentum: a roadmap to end viral hepatitis

The transformation of HCV treatment as a result of the advent of direct-acting antivirals has infused the global hepatitis fight with renewed energy and momentum. As a result of sustained advocacy, the global community has come to recognize viral hepatitis as a critical global health priority. Through the global health sector strategy for viral hepatitis 2016–2021, countries have committed to take the action needed to halt the rise in mortality related to viral hepatitis and to put the world on track to end viral hepatitis as a public health threat. The global health sector strategy for viral hepatitis 2016–2021 outlines a series of concrete actions to fully leverage effective methods to prevent all forms of viral hepatitis and to scale up testing and treatment for HBV and HCV.

To apply these strategic directions in the WHO region with the heaviest hepatitis burden, South-East Asia is implementing its own regional action plan on viral hepatitis. The regional action plan outlines specific steps and targets to fully leverage strategic information for focused action, bring prevention and treatment interventions to those who need them, ensure equitable access, mobilize sufficient and sustainable financing, and catalyse innovation to accelerate gains. Together, the global strategy and the regional action plan provide an actionable framework for evidence-based, prioritized action to turn the tide against viral hepatitis.

## Challenges to hepatitis elimination

Scientific advances and new political momentum have generated optimism in the global response to viral hepatitis. However, reaching the goal of ending viral hepatitis as a public health threat will demand that persistent challenges to progress are confronted and overcome.

Weak health systems undermine hepatitis prevention efforts. As HCV transmission primarily occurs through unsafe blood or injections, simple infection control procedures, if mainstreamed and enforced across health systems, have the potential to sharply lower HCV incidence. However, important parts of the prevention agenda for viral hepatitis require action that extends well beyond the health sector. For example, access to safe water supplies and sanitation are critical to the prevention of hepatitis A and E.

The lack of clear, reliable evidence on the prevalence of hepatitis and the distribution of viral hepatitis among subpopulations in South-East Asia impedes the development and monitoring of sound national plans to fight viral hepatitis. In contrast to the HIV response, which benefited from strong to sustained donor support, no dedicated, catalytic funding source is readily available for hepatitis prevention and treatment services. In part due to the low priority accorded to viral hepatitis by leading donors, prevention and treatment of hepatitis persist as low political priorities in many countries.

The drugs historically available to treat viral hepatitis also slowed progress, due to their limited effectiveness and to their high prices. The emergence of affordable, highly effective, easier-to-take direct-acting antivirals offers the possibility of transcending the inherent limitations of older drugs. In the case of HBV, however, treatment regimens are complex and lifelong, posing challenges to efforts to reduce HBV-related mortality.

## Translating rhetoric into action: key actions steps

Although international donors have played a key role in the progress made in recent years against HIV, TB and Malaria, the truth is that national governments have largely driven efforts that have reversed these epidemics. Similarly, in the case of viral hepatitis, national governments must own and lead the development of evidence-based policies and programmes to sharply lower morbidity and mortality associated with viral hepatitis. National governments should leverage progress towards universal health coverage to ensure that the response to viral hepatitis is equitable and grounded in a respect for human rights. While national governments must lead the fight against hepatitis, they cannot conquer viral hepatitis on their own, underscoring the urgent need to partner and collaborate with communities, people living with viral hepatitis, and other key sectors.

**Fig. 3:**
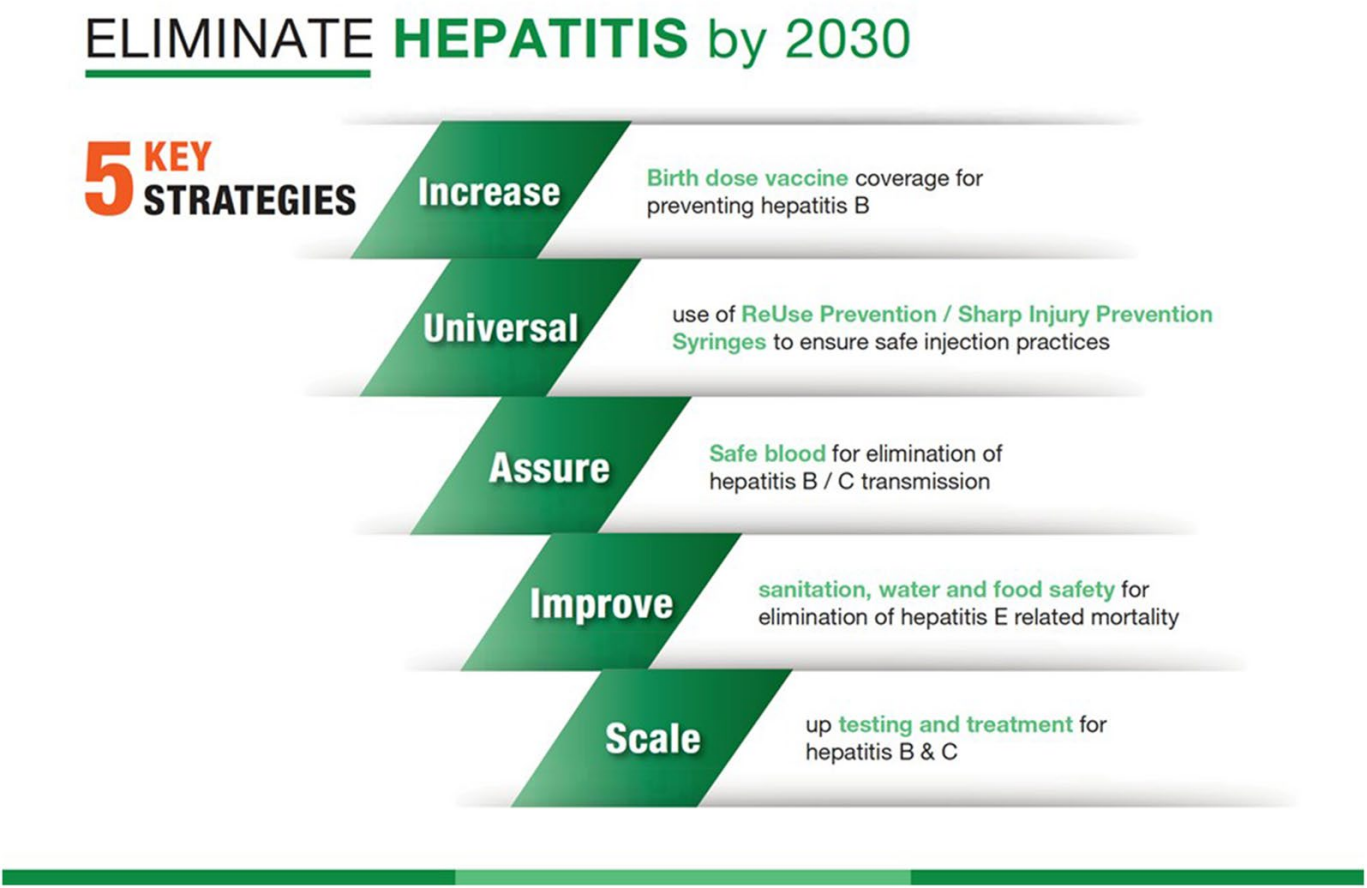
Five key interventions for hepatitis elimination (Source: Hepatitis Advocacy Document prepared for the 17th session of the WHO Regional Committee for South-East Asia, September 2017)

Several key actions are needed (Fig. 3). First, all infants and newborns must be immunized at birth for HBV, with an additional two to three doses required as follow-up, depending on national guidelines. At no more than 50 cents per child, the HBV vaccine saves a lifetime of health risks, including cirrhosis and liver cancer. Although the three-dose regimen has moderately high coverage (87%) in South-East Asia, only 34% of children in the region are immunized at birth [8].

In addition, injection safety in health settings must be ensured, including through the use of safe syringes that prevent re-use and sharp injury. As in the case of immunization, costs associated with injection safety are minimal, as each safe syringe costs no more than 10 cents extra while minimizing transmission risks for HBV and HCV infection, both of which are costly, life-threatening conditions.

Blood safety must also be assured, by requiring the screening of every unit of blood and related products for hepatitis B and C to block a major route of hepatitis transmission.

Drinking water must be made safe, and all people should have access to effective sanitation. Not only is this approach one of the most effective prevention strategies for viral hepatitis, but it is also a basic human right.

Finally, immediate steps are needed to scale up diagnostic and treatment facilities. Persons infected with Hepatitis B or C virus are usually unaware of their infection. Effective treatments exist for viral hepatitis at reasonable cost—under US$ 160 for a curative regimen for HCV and US$ 250 for a year’s worth of treatment for HBV [9]. Yet, despite the affordability of these life-saving regimens, few patients in the region—3% for people with HBV infection, and 9% of people with HCV—currently have access to testing and treatment services [9].

Failure to ensure access to effective diagnostic and therapeutic tools contributes to substantial, preventable illness and mortality associated with cirrhosis and liver cancer. It also wastes precious financial resources. Direct-acting antivirals for treatment of HCV are cost-effective within 2 years of treatment and cost-saving within 10 years of treatment.

## Conclusion

We have the tools and the knowledge we need to prevent morbidity and mortality associated with viral hepatitis—including universal vaccination of newborns and infants, which would have the greatest impact on new HBV infections, as well as treatments for HBV and HCV that are now affordable. Now we must act to put these tools and knowhow into action. If we do not, we will miss the chance to end viral hepatitis by 2030.

Thus far, political commitment has been the key missing ingredient in the fight against viral hepatitis. With momentum from newer scientific breakthroughs, global commitments and reductions in the prices of key medicines, much stronger political action is needed to take the hepatitis response to the next level. National action plans must be fully funded and implemented, and political leaders need to actively engage physicians’ associations, academics, patient support groups, affected populations, non-governmental organizations, the private sector, media and celebrities to build awareness of the hepatitis challenge and mobilize diverse sectors around the common goal of ending hepatitis.
